# Assessment of inhaled corticosteroids use and associated factors among asthmatic patients attending Tikur Anbessa Specialized Hospital, Ethiopia

**DOI:** 10.1186/s13104-017-2645-2

**Published:** 2017-07-25

**Authors:** Yohanes Ayele, Ephrem Engidawork, Tola Bayisa

**Affiliations:** 1 Department of Clinical Pharmacy, School of Pharmacy, College of Health and Medical Sciences, Haramaya University, P.O. Box 235, Harar, Ethiopia; 2 Department of Pharmacology and Clinical Pharmacy, School of Pharmacy, College of Health Sciences, Addis Ababa University, Addis Ababa, Ethiopia; 3 Department of Internal Medicine, School of Medicine, College of Health Sciences, Addis Ababa University, Addis Ababa, Ethiopia

**Keywords:** Asthma, Persistent, Inhaled corticosteroids, Underutilization

## Abstract

**Background:**

Inhaled corticosteroids (ICSs) are cornerstone therapy for persistent asthma. However, underutilization of ICSs is common and little is known about factors contributing toward this undesirable use.

**Methods:**

A cross-sectional study was conducted through interview and chart review among persistent asthmatic patients attending chest clinic of Tikur Anbessa Specialized Hospital from 1 May to 31 September 2014. A total of 131 eligible patients who attended the clinic during study period were included in the study. A multivariate logistic regression was used to examine the association between independent and dependent variables.

**Results:**

Overall, extent of underutilization of ICSs was found to be 68%. Monthly income, comorbidity and types of ICSs products prescribed showed significant association with reported underutilization. Patients’ reported reasons for underutilization includes; unaffordability (44%), using only when symptoms exacerbate (21%), fear of side effects (10%), practitioners’ recommendation (10%) and unavailability of ICSs in the local market (7%). Physicians also stated unaffordability, fear of side effects and dependency, lack of local guideline for asthma management and unavailability of ICSs as the contributing factors.

**Conclusion:**

In this setting, extent of underutilization of ICSs was found to be high and seems the result of complex interaction of various factors. Financial problem combined with inconsistent availability of ICSs in the local market, patients’ poor knowledge of asthma and ICSs, negative attitude toward ICSs, absence of local guidelines for asthma management are found to be essential elements dictating an extent of ICSs use.

**Electronic supplementary material:**

The online version of this article (doi:10.1186/s13104-017-2645-2) contains supplementary material, which is available to authorized users.

## Background

The incidence of asthma has increased dramatically in the past few decades. According to Global Asthma Network report [Global Initiative for Asthma (GINA)], approximately 334 million people worldwide have asthma with high burden of disability [1]. The historic perception of associating asthma with developed countries no longer exist as rapidly increasing number of people are being affected by the disease in developing countries. The mean prevalence of diagnosed asthma was highest in the richest countries (9.4%) followed closely by the poorest countries (8.2%). The middle-income countries had the lowest prevalence (5.2%) [2].

Airway inflammations are a major factor in the pathophysiology of asthma and remain as a principal target of treatment. Although asthma cannot be cured, appropriate management can ensure adequate control of the disease, reverse and even prevent disease process and enable people to have good quality of life [3].

Pharmacological therapy is a key part of asthma management. Generally, asthma drugs can be classified as relievers and controller. There are two groups of controllers; those with anti-inflammatory action including corticosteroids and leukotriene modifiers, and those with a sustained bronchodilator action including long acting β-agonists (LABAs) and slow-releasing theophylline [3, 4]. Medications with anti-inflammatory activity are recommended for all patients with persistent asthma. Inhaled corticosteroids (ICSs) are the most effective medications for long-term control of persistent asthma across all age groups and in most of the therapy care steps [5]. However, studies done in different part of the world indicate low utilization of these key medications, developing countries suffering the most [6–9]. Given the importance of this medication, it would be crucial to determine causes of such underuse. Therefore, the aim of this study was to identify the factors associated with underutilization of ICS among persistent asthma patients in Tikur Anbessa Specialized Hospital (TASH).

## Methods

A prospective cross-sectional study was conducted at the chest clinic of TASH from 1 May to 31 September 2014. TASH is located in Addis Ababa, the capital of Ethiopia, and it is the largest hospital in the country providing a tertiary level care.

All patients with persistent asthma of duration greater than 1 year were included. Patients were excluded if they were <18 years of age or had one or more of the following comorbidities: congestive heart failure, other lung disease [including but not limited to chronic obstructive pulmonary diseases (COPDs), cystic fibrosis, and interstitial lung disease], or other medical conditions, such as severe anemia or malignancy, that could have made categorization of asthma severity difficult.

All asthmatic patients who attended the clinic for their regular follow-up during study period and fulfilled inclusion criteria were included in the study summing up a total of 131 patients. Eight physicians (four fellows and four residents) working at the chest clinic were requested to fill semi-structured questionnaires for qualitative data.

Data were collected through patient interview using a semi-structured questionnaire comprising socio-demographic characteristics, disease characteristics, ICS use, and knowledge and attitude towards asthma and ICS. The questionnaires were adapted from previous studies and appropriate modifications were made to serve our purposes [10, 11]. In addition, the charts of the patients were also reviewed particularly to generate information pertaining to medication history. Data from physicians was collected by using self-administered semi-structured questionnaire focusing their perspectives and experiences on patients’ ICS use. Severity of asthma was determined by using GINA criteria [2] such as frequency of symptoms in a week, frequency of nighttime awakening symptoms in a past month, interference with normal activity, the use short acting β_2_ agonist and lung function. Accordingly, mild persistent asthma was defined as occurrence of symptoms >2 days/week but not daily or occurrence of nighttime awakening symptoms three to four times a month or the use of short acting β_2_ agonist >2 days/week but not daily, and not more than 1× on any day with minor limitation and forced expiratory volume in 1 s (FEV_1_) >80% predicted. Moderate persistent asthma was defined as occurrence of symptoms daily or occurrence of nighttime awakening symptoms >1×/week but not nightly or the use of short acting β_2_ agonist daily with some limitation and FEV_1_ >60% but <80% predicted. Severe persistent asthma was defined as occurrence of symptoms throughout the day or occurrence of nighttime awakening symptoms nightly or the use of short acting β_2_ agonist several times per day with extreme limitation and FEV_1_ <60% predicted.

The regimen of ICS recommended for the patient is reviewed from the chart prior to the interview. Patients were also requested to report the regimen they have been using with the type of product prescribed. The extent of underutilization of ICS was calculated considering the appropriateness of ICS regimen (the dose and frequency of ICS) the patients have been using for given asthma category compared against recommendation of GINA guideline for the specific step of asthma management [1]. In addition, patients who were eligible for ICS but without prescription for ICS, patients who were prescribed ICS but not using then were counted under category of inadequate ICS use. Asthma and ICS knowledge was dichotomized into good and poor by computing sum of questionnaires assessing knowledge for each patient and taking median score as cut-off point. Similarly, attitude toward asthma and ICS were dichotomized into positive and negative attitude by computing sum of the questionnaires assessing attitude for each patient and taking the median score as cut-off point.

The data was entered into epi info version 7 and analyzed using Statistical Package for Social Science (SPSS) for windows version 20. Each variable was evaluated independently in a bivariate analysis and association was determined using cross tabulation and crude odds ratio (COR) with 95% CI. All variables associated with the underutilization of ICS at a probability level of less than or equal to 0.25 on the bivariate analysis were entered into a multivariate logistic regression analysis to control for confounders. The covariates tested includes; educational status, types of ICS products prescribed, monthly income, duration of asthma, comorbidity, severity of asthma, asthma knowledge and ICS knowledge. The final model was selected by using Wald criterion and Hosmer and Lemeshow test to determine variables that were independently associated with underutilization of ICS at a probability level of less than 0.05 and presented using adjusted odds ratio (AOR).

## Operational definition

### Inhaled corticosteroids

Asthma medication products containing either corticosteroid alone or in combination with LABAs.

### Underutilization of inhaled corticosteroids

The use of suboptimal regimen compared against the recommendation by GINA guideline for the specific step of asthma management or primary non-adherence or discontinuation in all or not having prescription for ICSs in case of persistent asthma patients.

### Primary non-adherence

Patients who were prescribed ICSs but never filled the prescription.

## Results

### Socio-demographic characteristics of participants

A total of 131 subjects were included in the final analysis. The mean age of the study subjects was 52.29. Significant proportions of study population were women (64%) and considerable (65%) numbers of the participants were married. With regards to educational status, a sizable proportion (34.4%) of the patients had college diploma and above, while 25.2% of the participants were not fortunate enough to have formal education. The majority (73%) of study subjects were earning less than 1200 Ethiopian Birr (ETB) which is equivalent to $53.5 on monthly basis considering all sources of their income.

### Disease characteristics of the asthmatic patients

The mean duration of asthma for this study group during study period was 20.9 years and more than half (52%) of study participants had asthma of greater than 21 years duration. The severity category of asthma was found to be distributed roughly equal among all categories: 34.4, 33.6 and 32.1% of the participants had mild persistent asthma, moderate persistent asthma and severe persistent asthma, respectively. About 59% of the respondents had one or more comorbid condition and under half (43.5%) of the study population were on one or more medications other than anti-asthmatics. The common comorbidities reported in these participants were gastro-esophageal reflux disorder (20.7%), hypertension (19%), diabetes mellitus (12%), allergic rhinitis (10%) and mental illness (10%). Other comorbidities such as sinusitis, heart failure and osteoarthritis were also reported.

### Inhaled corticosteroids use

As it can be seen from Table 1, ICS were prescribed in 90.8% of the cases; out of which corticosteroid products prescribed alone was accounted for about 74% of the cases. The remaining received combination product, corticosteroids with LABA. Among the patients prescribed with ICS, 61.3% had been taking ICS, 23.5% discontinued, and 15.1% were primary non-adherent. From those who had been taking ICS, about 42% of the patients were on suboptimal regimen. Overall underutilization of ICS was found to be 68%.

**Table 1:** Inhaled corticosteroids use in TASH chest clinic from 1 May to 31 September 2014 (n = 131)

ICS use	Number (%)
ICS prescribed	
Yes	119 (90.8)
No	12 (9.2)
Types of ICS products prescribed	
ICS alone	88 (73.9)
ICS with LABA	31 (26.1)
ICS prescribed patients (n = 119)	
Patients using ICS	73 (61.3)
Patients who discontinued using ICS	28 (23.5)
Primary non-adherence	18 (15.1)
Patients who were using ICS (n = 73)	
Patients on optimal regimen	42 (57.5)
Patients on suboptimal regimen	31 (42.5)
Underutilization of ICS	
Yes	89 (68)
No	42 (32)

*TASH* Tikur Anbessa Specialized Hospital

### Patients’ knowledge and attitude toward asthma and Inhaled corticosteroids

Table 2 shows that more than half (61.4%) of the ICS users had good asthma knowledge, while most of them (90.1%) had positive attitude towards asthma. Approximately 46% of the ICS users had good knowledge of ICS and only 54.5% of the ICS users displayed positive attitude toward ICS.

**Table 2:** Patients’ knowledge and attitude toward asthma and inhaled corticosteroids in TASH chest clinic from 1 May to 31 September 2014 (n = 131)

Knowledge and attitude toward asthma and ICS	Number (%)
Asthma knowledge	
Good	77 (61.4)
Poor	54 (38.6)
Attitude towards asthma	
Positive	106 (90.1)
Negative	25 (9.9)
ICS knowledge (n = 119)^a^	
Good	55 (45.5)
Poor	64 (54.5)
Attitude toward ICS (n = 119)	
Positive	64 (54.5)
Negative	55 (45.5)

*TASH* Tikur Anbessa Specialized Hospital

^a^Patients filled with ICS only were assessed for ICS knowledge and attitude

### Factors associated with underutilization of inhaled corticosteroids

Table 3 illustrates a result of multivariate logistic regression analysis of factors associated with underutilization of ICS among study subjects. It shows that patients who earned monthly income less than or equal to 1200 ETB were about five times more likely to underutilize ICS than those earned greater than 2500 ETB [95% CI 1.10–22.16]. Patients with comorbidity were about three times more likely to underutilize ICS than those with no comorbidity [95% CI 1.11–8.69]. The model also indicated 4.4 times increased odds of underutilization among patients who had been prescribed with ICS alone compared to patients who had been filled with combination products [95% CI 1.49–12.92].

**Table 3:** Multivariate analysis of factors associated with underutilization of inhaled corticosteroids in TASH chest clinic from 1 May to 31 September 2014 (n = 131)

Variables	Underutilization of ICS		COR (95% CI)	AOR
	Yes n (%)	No n (%)		
Monthly income in ETB (=120)^a^				
≤1200	56 (78.3)	17 (21.7)	*8.10 (2.19–30.00)* ^a^	*5.35 (1.10–22.16)*
1201–2500	15 (51.9)	13 (48.1)	2.42 (0.59–9.82)	1.46 (0.29–7.22)
>2500	10 (30.8)	9 (69.2)	1.01	1.00
Duration of asthma in years				
≤21	36 (56.9)	28 (43.1)	1.00	1.00
>21	47 (72.1)	20 (27.9)	1.96 (0.91–4.21)	1.49 (0.54–4.15)
Comorbidity				
Yes	54 (71.8)	23 (28.2)	*2.15 (1.001–4.65)* ^a^	*3.10 (1.11–8.69)* ^a^
No	29 (54.2)	25 (45.2)	1.00	1.00
Types of ICS products prescribed				
ICS alone	68 (73.9)	26 (26.1)	*4.44 (1.88–10.62)* ^a^	*4.39 (1.49–12.92)* ^a^
ICS with LABA	15 (38.7)	21 (61.3)	1.00	1.00
Severity of asthma				
Mild	28 (61.9)	18 (38.1)	1.00	1.00
Moderate	23 (52.5)	21 (47.5)	0.68 (0.28–1.64)	0.545 (0.16–1.87)
Severe	32 (81.1)	9 (18.9)	2.63 (0.94–7.40)	3.17 (0.86–11.75)

Variables with significant association are indicated in italics

*TASH* Tikur Anbessa Specialized Hospital, *CI* confidence interval, *COR* crude odds ratio, *AOR* adjusted odds ratio

^a^ Remaining subjects were non respondents, Ethiopian per capita income is $590, price of beclate (beclomethasone) 200 μg in Ethiopia is 110 ETB

### Patients’ reasons for reported underutilization of inhaled corticosteroids

Patients (89) who deemed as under utilizers of ICS were asked about the factors contributing for underutilization and considerable portion (44%) mentioned unaffordability. In addition, about 21% of the patients tended to decrease the dose or change the dose frequency of the drug when they felt symptom free leading to use of suboptimal regimen, while about 10% of patients claimed that they were told to so by the practitioners themselves. Patients also referred side effects (10%) and unavailability of ICS (8%). Forgetfulness, lack of effect, not liking the drug, and fear of dependence are other reasons cited by the remaining respondents for reported underutilization.

### Physicians’ reported reasons for patients’ underutilization of inhaled corticosteroids

Eight physicians working in chest clinic during data collection period were requested to fill questionnaires regarding to adequacy of ICS utilization from patients’ side, almost all of the physicians participated in the study responded ‘no’ and cited different reasons for the problem. However, most of them (75%) stated cost of ICS as a major barrier. Physicians also pointed out patients’ perceptions such as fear of side effects (62.5%) and dependence (50%) as causes for underutilization. Some of the physicians (25%) also mentioned lack of standard of practice such as local guideline as a stumbling block for appropriate use of ICS.

Physicians were also probed if there were any circumstances in which they prescribed other drugs than ICS, all of them said ‘yes’ revealing the use of either oral steroids or theophedrine for uncontrolled asthma (75%). Fifty percent of physicians also pointed out other reason like unavailability of ICS in the local market and patients’ inability to afford ICS as a driving force in the drug selection process.

## Discussion

The primary objective of this study was to assess factors associated with underutilization of ICS among persistent asthma patients under regular follow up at the chest unit of TASH. In the present study, high prevalence of underutilization of ICS was found from patients’ perspective. Significant association was found between underutilization of ICS and patients with monthly income of less than 1200 ETB, comorbidity, and types of ICS prescribed. Commonly cited reasons for underutilization of ICS were increased cost, use only when symptoms appear, patients perception towards ICS and inconsistent availability.

Overall, in this study, underutilization of ICS was found to be 68%. The finding is in agreement with a study done in the US where 65% of the patients were using a dose of ICS below that recommended in the National Heart, Lung, and Blood Institute guideline for asthma diagnosis and management [12]. However, the major difference between these two studies is that in the later study, most of the underutilization appeared to represent physicians’ recommendations. This is not the case in this study where underutilization of ICS was primarily due to primary non-adherence, discontinuation, and suboptimal regimen use.

Generally, it is well understood that cost of medications affects utilization pattern of patients adversely. The problem is critical when it comes to asthma drugs, particularly ICS, which is the mainstay therapy of asthma. In the current study, monthly income of participants was significantly associated with underutilization of ICS. Though it is difficult to compare income pattern of different countries hence its association with underutilization of ICS, our finding is in agreement with studies done in elsewhere [13–15].

The present study also showed significant association between underutilization of ICS and comorbidity. Similar finding is reported form Canadian study [16]. Increased odds of underutilization of ICS among patients with one or more comorbidity could be due to different reasons. Patients with comorbidity are more likely to use other medications than ICS which may lead to increased financial burden. Relatedly, taking medications other than asthma medications can result in complicated and burdensome medication regimens often leading to forgetfulness.

In the present study, types of ICS product prescribed, whether corticosteroids were prescribed alone or in combination with LABA, was significantly associated with ICS underutilization. According to the guideline when corticosteroids alone is used, increasing the dose from low to moderate or high is recommended depending on the steps of management or severity of the disease. However, sometimes physicians fail to do so and tend to recommend similar dose across most steps of asthma management [17]. This malpractice could be associated with absence of local clinical guideline for management of asthma as it was mentioned by some of the practitioners in the setting. On the other hand, in spite of having prescription for combination therapy, patients tend to use corticosteroids alone mostly due to increased cost of combination therapy which might be inadequate (in terms of dose and frequency) for the given step of management.

With respect to patients’ reported reasons for underutilization of ICS, unaffordability of ICS was found to have big influence on ICS use (44%). This finding is in line with reports from elsewhere [16–20]. In addition, unavailability of ICS in the study setting was reported from both patients and care providers as one of the factors contributing for underutilization of ICS.

Another frequently stated reason in association with underutilization of ICS was use of ICS when only symptoms appear (21%). Although there was no significant association found between ICS underutilization and knowledge of ICS and asthma, in this study, only 45.5 and 64.1% of the patients had good knowledge of ICS and asthma, respectively. This finding supplements the idea that patients’ tendency to use ICS when symptoms appears is because of lack of understanding about the nature of the disease, and exact role ICS in asthma therapy as well as the necessity for their continuous use as it is indicated in other studies [21, 22].

In this study, about 9% of patients with persistent asthma had not received prescription for ICS which is in consistent with other studies [21, 23]. Aforementioned under prescription could be attributed to different reasons. Firstly, physician may prescribe other drug than ICS intentionally when the patients fail to afford ICS as it was reported by some of the physicians. Secondly, physicians also tend to use drugs from other class for uncontrolled/refractory asthma management which might have contributed for reported under prescribing.

Finally, in this study, absence of local protocol for management of asthma was shown to have negative impact on the proper utilization of ICS from physicians’ side. Although physicians are expected to use international guidelines in this situation, inconsistent prescribing pattern and failure to adhere to such guidelines might occur [23].

## Conclusion

In the present study, extent of underutilization of ICS was found to be high amid various factors. Financial problem combined with inconsistent availability of ICS in the local market, poor patients’ knowledge of asthma and ICS, negative attitude toward ICS, absence of local guidelines for asthma management are shown to be major contributing factors for the reported underutilization of ICS. Therefore, concerned bodies should ensure consistent availability and affordability of ICS. It is also very essential to increase awareness of the patients on asthma and ICS and to prepare local guideline for management of asthma.

## Limitation of the study

Since it is a cross-sectional design, it was not possible to find temporal relationship between cause and effect. The present study had also limitation regarding its generalizability. First, the study was not multi-centered, hence did not address different population of asthma. Secondly, due to poor patients flow during study period it was not possible to attain maximum sample size. Therefore, this result should be extrapolated cautiously.

## Additional file

**Additional file 1.** The data collection tools (Interview questionnaires for patients and self-administered questionnaires).

## Abbreviations

AOR: adjusted odds ratio
AAU: Addis Ababa University
COPD: chronic obstructive pulmonary diseases
COR: crude odds ratio
ETB: Ethiopian Birr
FEV_1_: forced expiratory volume in 1 s
GINA: Global Initiative for Asthma
ICS: inhaled corticosteroids
LABA: long acting β_2_-agonists
TASH: Tikur Anbessa Specialized Hospital
SPSS: Statistical Package for Social Science
