# Supplementary material for: Assessment of inhaled corticosteroids use and associated factors among asthmatic patients attending Tikur Anbessa Specialized Hospital, Ethiopia
Source: BMC Res Notes. 2017 Jul 25;10:314. doi: 10.1186/s13104-017-2645-2 (PMC5526241; doi:10.1186/s13104-017-2645-2)
Supplement: Supplementary file 1 — Additional file 1. The data collection tools (Interview questionnaires for patients and self-administered questionnaires). [file 13104_2017_2645_MOESM1_ESM.docx]

**Annex I**

**Participants Information Sheet and Informed Voluntary Consent Form for Participants Selected for the Study**

My name is_____________________ I am working as data collector for the study being conducted in this Hospital by Addis Ababa University; I kindly request you to lend me your attention to explain you about the study.

**Study Title:** Assessment of Inhaled Corticosteroids Use and Associated Factors among Asthmatic Patients Attending Tikur Anbessa Specialized Hospital, Ethiopia

**Purpose:-**The study will be helpful to assess Inhaled Corticosteroids Use associated factors generating information for future action.

**Procedure and duration:** There are about 35 questions to be answered where I fill the questioner by interviewing you. The interview will take around 20 minutes.

**Risks and benefits: -** The risk of being participating in this study is very minimal, except taking some minutes, for the interview, there is no other risk that you will experienced. By participating in this study you will not get direct benefit but the outcome of this study will be useful tool for decision makers

**Confidentiality: -** The information, which you provide us, will be confidential. There will be no information that will identify you. The finding of study will be general for the study population and will not reflect any thing particular of individual person. The questionnaire will be coded to exclude showing names; no references will be made in oral or written reports that could link participants to the research.

**Right: -** Participation in this study is voluntary. You have the right to declare not to participate in this study. If you decide to participate first, you have the right to with draw from the study at any time and this will not result in you for any loss of benefit, which you otherwise are entitled. You do not have to answer any question that you do not want to answer.

**Contact address:-**If you have any questions or enquiries at any time about the study or the procedures, please contact and communicate with Principal investigator:-Mr. YOHANES AYELE :- +251920297839, [yohanesayele@ymail.com](mailto:yohanesayele@ymail.com),

**Declaration of informed voluntary Consent:**

I have clearly understood the purpose of the research, the procedure, risks and benefits, issues of confidentiality rights of participating and contact address for any queries. I have given the opportunity to ask questions for things that may have been unclear. I was informed that I have the right to with draw from the study at any time; therefore I declare my voluntary consent to participate in this study with my signature.

Name and Signature of Participants: _____date_____/_____/2014/15

Name and Signature of data collector: ___date_____/_____/2014/15

**Participant interview questionnaires**

| **Section** **I. Socio-demographic data[** | | | | | | | | | | **Remark** | |  |  |
| --- | --- | --- | --- | --- | --- | --- | --- | --- | --- | --- | --- | --- | --- |
| 500 | Age | | | | ______(years) | | | | |  | |  |  |
| 501 | Sex | | | | 1-Male | | | | |  | |  |  |
|  |  |  |  |  | 2-Female | | | | |  |  |  |  |
| 502 | Marital status | | | | 1-Single | | | | |  | |  |  |
|  |  |  |  |  | 2-Married | | | | |  |  |  |  |
|  |  |  |  |  | 3-Divorced | | | | |  |  |  |  |
|  |  |  |  |  | 4-Widow | | | | |  |  |  |  |
| 503 | The highest education achieved | | | | 1-No formal education | | | | |  | |  |  |
|  |  |  |  |  | 2-Primary education | | | | |  |  |  |  |
|  |  |  |  |  | 3-High school | | | | |  |  |  |  |
|  |  |  |  |  | 4-Diploma and above | | | | |  |  |  |  |
| 504 | Monthly income | | | | _______(ETB) | | | | |  | |  |  |
| **Section** **II. Disease characteristics** | | | | | | | | | | | |  |  |
| 600 | How long is it since you have been diagnosed with asthma? | | | | | ______(years) | | | |  | |  |  |
| 601 | In the past 1 weeks, how many days have you had problems with coughing, wheezing, shortness of breath, or chest tightness during the day? | | | | | _____(days) | | | |  | |  |  |
| 602 | In the past 1 month, how many days have you awakened at night from sleep because of coughing or other asthma symptoms? | | | | | _____(days) | | | |  | |  |  |
| 603 | In the past 1 month how many days have you had symptoms while exercising or playing | | | | | _____(days) | | | |  | |  |  |
| 604 | How many puffs of your (quick-relief medicine) do you use per day? | | | | | ______per day | | | |  | |  |  |
| 605 | How many (name short-acting inhaled beta2-agonist) inhalers have you been through in the past month? | | | | | __________ | | | |  | |  |  |
| 606 | Have you tried any other medicines or remedies? | | | | | 1-Yes  2-No | | | |  | |  |  |
| 607 | Do you have any physician confirmed comorbidity? | | | | | 1. Yes 2. 2-No | | | | If the answer is NO go to Q 700 | |  |  |
| 608 | Please specify the disease you diagnosed with | | | | | 1________________  2________________  3________________ | | | |  | |  |  |
| 6099 | Do you use medication other than asthma medication on regular basis? | | | | | 1-Yes  2-No | | | |  | |  |  |
| **Section** **III. ICS use** | | | | | | | | | | | |  |  |
| 700 | Did you have prescription for ICS? | | | | 1-Yes | | | | | | If the answer is NO go to section IV |  |  |
|  |  |  |  |  | 2-No | | | | | |  |  |  |
| 701 | Please specify the product prescribed for you | | | |  | | | | | |  |  |  |
| 702 | Are you using ICS currently? | | | | 1-Yes | | | | | | If answer is ‘Yes’ go to Q703,  If answer is ‘discontinued’ go to Q704,  If answer is ‘never used’ go to Q705 |  |  |
|  |  |  |  |  | 2-Discontinued | | | | | |  |  |  |
|  |  |  |  |  | 3-Never used before | | | | | |  |  |  |
| 703 | Please tell me the dose and frequency you have been using | | | | Dose_______________  Frequency(per day)_________________ | | | | | |  |  |  |
| 704 | Please tell me why you did discontinue use of ICS | | | | 1-My symptom resolved | | | | | |  |  |  |
|  |  |  |  |  | 2- Because of side effects | | | | | |  |  |  |
|  |  |  |  |  | 3-Drug not affordable | | | | | |  |  |  |
|  |  |  |  |  | 4-Drug not available | | | | | |  |  |  |
|  |  |  |  |  | 5-Lack of effect | | | | | |  |  |  |
|  |  |  |  |  | 6-other_______________ | | | | | |  |  |  |
| 705 | Please tell me why you did never used the prescribed ICS | | | | 1-Drug not affordable | | | | | |  |  |  |
|  |  |  |  |  | 2-Drug not available | | | | | |  |  |  |
|  |  |  |  |  | 3-Do not like the drug | | | | | |  |  |  |
|  |  |  |  |  | 4-Other (specify) | | | | | |  |  |  |
| **Section IV. Asthma knowledge and attitude** | | | | | | | | | | | | |  |
| 800 | | Asthma is a chronic disease. | | | | | | 1-Yes | | | | |  |
|  |  |  |  |  |  |  |  | 2-No | | | | |  |
|  |  |  |  |  |  |  |  | 3-Don’t know | | | | |  |
| 801 | | The underlying cause of asthma symptoms is airway inflammation and  swelling. | | | | | | 1-Yes | | | | |  |
|  |  |  |  |  |  |  |  | 2-No | | | | |  |
|  |  |  |  |  |  |  |  | 3-Don’t know | | | | |  |
| 802 | | Asthma can kill. | | | | | | 1-Yes | | | | |  |
|  |  |  |  |  |  |  |  | 2-No | | | | |  |
|  |  |  |  |  |  |  |  | 3-Don’t know | | | | |  |
| 803 | | It is possible to prevent asthma symptoms (wheezing, cough, chest  tightness) by appropriate medication. | | | | | | 1-Yes | | | | |  |
|  |  |  |  |  |  |  |  | 2-No | | | | |  |
|  |  |  |  |  |  |  |  | 3-Don’t know | | | | |  |
| 804 | | It is important to take some asthma medication even though there is no  symptom. | | | | | | 1-Yes | | | | |  |
|  |  |  |  |  |  |  |  | 2-No | | | | |  |
|  |  |  |  |  |  |  |  | 3-Don’t know | | | | |  |
| **Attitude toward asthma** | | | | | | | | | | | | |  |
| 805 | | Asthma is serious disease and must take treatment. | | | | | | 1-Strongly disagree | | | | |  |
|  |  |  |  |  |  |  |  | 2-Disagree | | | | |  |
|  |  |  |  |  |  |  |  | 3- Neutral | | | | |  |
|  |  |  |  |  |  |  |  | 4- Agree | | | | |  |
|  |  |  |  |  |  |  |  | 5-Strongly agree | | | | |  |
| 806 | | My asthma would be worse if I did not take medication at all. | | | | | | 1-Strongly disagree | | | | |  |
|  |  |  |  |  |  |  |  | 2-Disagree | | | | |  |
|  |  |  |  |  |  |  |  | 3- Neutral | | | | |  |
|  |  |  |  |  |  |  |  | 4-Agree | | | | |  |
|  |  |  |  |  |  |  |  | 5-Strongly agree | | | | |  |
| 807 | | I believe my asthma medication will control my asthma. | | | | | | 1-Strongly disagree | | | | |  |
|  |  |  |  |  |  |  |  | 2-Disagree | | | | |  |
|  |  |  |  |  |  |  |  | 3- Neutral | | | | |  |
|  |  |  |  |  |  |  |  | 4- Agree | | | | |  |
|  |  |  |  |  |  |  |  | 5-Strongly agree | | | | |  |
| 808 | | All my asthma medication is important for me. | | | | | | 1-Strongly disagree | | | | |  |
|  |  |  |  |  |  |  |  | 2-Disagree | | | | |  |
|  |  |  |  |  |  |  |  | 3- Neutral | | | | |  |
|  |  |  |  |  |  |  |  | 4- Agree | | | | |  |
|  |  |  |  |  |  |  |  | 5-Strongly agree | | | | |  |
| 809 | | I do not feel comfortable when people know that I am asthmatic patient. | | | | | | 1-Strongly disagree | | | | |  |
|  |  |  |  |  |  |  |  | 2-Disagree | | | | |  |
|  |  |  |  |  |  |  |  | 3- Neutral | | | | |  |
|  |  |  |  |  |  |  |  | 4-Agree | | | | |  |
|  |  |  |  |  |  |  |  | 5- Strongly agree | | | | |  |
| 810 | | I do not fear taking asthma medications in front of peoples. | | | | | | 1-Strongly disagree | | | | |  |
|  |  |  |  |  |  |  |  | 2-Disagree | | | | |  |
|  |  |  |  |  |  |  |  | 3- Neutral | | | | |  |
|  |  |  |  |  |  |  |  | 4- Agree | | | | |  |
|  |  |  |  |  |  |  |  | 5-Strongly agree | | | | |  |
| **Section V. ICS Knowledge and attitude** | | | | | | | | | | | | |  |
| 900 | | | ICS is drug of choice for long term prevention of asthma. | | | | | | 1-Yes | | | |  |
|  |  |  |  |  |  |  |  |  | 2-No | | | |  |
|  |  |  |  |  |  |  |  |  | 3-Don’t know | | | |  |
| 901 | | | ICS work by fighting inflammation. | | | | | | 1-Yes | | | |  |
|  |  |  |  |  |  |  |  |  | 2-No | | | |  |
|  |  |  |  |  |  |  |  |  | 3-Don’t know | | | |  |
| 902 | | | The full effect of ICS depends on regular use. | | | | | | 1-Yes | | | |  |
|  |  |  |  |  |  |  |  |  | 2-No | | | |  |
|  |  |  |  |  |  |  |  |  | 3-Don’t know | | | |  |
| 903 | | | ICS may take days to produce full effect. | | | | | | 1-Yes | | | |  |
|  |  |  |  |  |  |  |  |  | 2-No | | | |  |
|  |  |  |  |  |  |  |  |  | 3-Don’t know | | | |  |
| 904 | | | When you use ICS, your wheeze or chest tightness gets better  immediately. | | | | | | 1-Yes | | | |  |
|  |  |  |  |  |  |  |  |  | 2-No | | | |  |
|  |  |  |  |  |  |  |  |  | 3-Don’t know | | | |  |
| **Attitude toward ICS** | | | | | | | | | | | | | |
| 905 | | | | | Asthmatic patients should use prophylactic treatment even if they are  feeling well. | | | 1-Strongly disagree | | | | | |
|  |  |  |  |  |  |  |  | 2-Disagree | | | | | |
|  |  |  |  |  |  |  |  | 3-Neutral | | | | | |
|  |  |  |  |  |  |  |  | 4-Agree | | | | | |
|  |  |  |  |  |  |  |  | 5-Strongly agree | | | | | |
| 906 | | | | | Regular use of ICS is important to prevent asthma attack and hospitalization. | | | 1-Strongly disagree | | | | | |
|  |  |  |  |  |  |  |  | 2-Disagree | | | | | |
|  |  |  |  |  |  |  |  | 3-Neutral | | | | | |
|  |  |  |  |  |  |  |  | 4-Agree | | | | | |
|  |  |  |  |  |  |  |  | 5-Strongly agree | | | | | |
| 907 | | | | | Since I have been using ICS, my asthma has gotten better. | | | 1-Strongly disagree | | | | | |
|  |  |  |  |  |  |  |  | 2-Disgree | | | | | |
|  |  |  |  |  |  |  |  | 3-Neutral | | | | | |
|  |  |  |  |  |  |  |  | 4-Agree | | | | | |
|  |  |  |  |  |  |  |  | 5-Strongly agree | | | | | |
| 908 | | | | | Regular use of ICS result dependency. | | | 1-Strongly disagree | | | | | |
|  |  |  |  |  |  |  |  | 2-Disagree | | | | | |
|  |  |  |  |  |  |  |  | 3-Neutral | | | | | |
|  |  |  |  |  |  |  |  | 4-Agree | | | | | |
|  |  |  |  |  |  |  |  | 5-Strongly agree | | | | | |
| 909 | | | | | Regular use of ICS can cause side effects | | | 1-Strongly disagree | | | | | |
|  |  |  |  |  |  |  |  | 2-Disagree | | | | | |
|  |  |  |  |  |  |  |  | 3-Neutral | | | | | |
|  |  |  |  |  |  |  |  | 4-Agree | | | | | |
|  |  |  |  |  |  |  |  | 5-Strongly agree | | | | | |

Name of data collector:_________________________________________Signature______

Name of Supervisor:___________________________________________Signature________

Date:­­­­_______________________________

**Thank You!!!**

**Annex II**

**Participants Information Sheet and Informed Voluntary Consent Form for physicians participated in the Study**

My name is_____________________ I am working as data collector for the study being conducted in this Hospital by Addis Ababa University; I kindly request you to lend me your attention to explain you about the study.

**Study Title:** Assessment of Inhaled Corticosteroids Use and Associated Factors among Asthmatic Patients Attending Tikur Anbessa Specialized Hospital, Ethiopia

**Purpose:-**The study will be helpful to assess Inhaled Corticosteroids Use associated factors generating information for future action.

**Procedure and duration:** There are about 8 questions to be answered where yo fill the answer in given space. The self-administered questionnaires will take around 10 minutes.

**Risks and benefits: -** The risk of being participating in this study is very minimal, except taking some minutes, for the interview, there is no other risk that you will experienced. By participating in this study you will not get direct benefit but the outcome of this study will be useful tool for decision makers

**Confidentiality: -** The information, which you provide us, will be confidential. There will be no information that will identify you. The finding of study will be general for the study population and will not reflect any thing particular of individual person. The questionnaire will be coded to exclude showing names; no references will be made in oral or written reports that could link participants to the research.

**Right: -** Participation in this study is voluntary. You have the right to declare not to participate in this study. If you decide to participate first, you have the right to with draw from the study at any time and this will not result in you for any loss of benefit, which you otherwise are entitled. You do not have to answer any question that you do not want to answer.

**Contact address:-**If you have any questions or enquiries at any time about the study or the procedures, please contact and communicate with Principal investigator:-Mr. YOHANES AYELE :- +251920297839, [yohanesayele@ymail.com](mailto:yohanesayele@ymail.com),

**Declaration of informed voluntary Consent:**

I have clearly understood the purpose of the research, the procedure, risks and benefits, issues of confidentiality rights of participating and contact address for any queries. I have given the opportunity to ask questions for things that may have been unclear. I was informed that I have the right to with draw from the study at any time; therefore I declare my voluntary consent to participate in this study with my signature.

Name and Signature of Participants: _____date_____/_____/2014/15

Name and Signature of data collector: ___date_____/_____/2014/15

**Self-administered Questionnaires for data collection from physicians**

1. What is your choice of drug for treatment of persistent asthma?

1-ICS

2-ICS+LABA

3-Other drugs (specify please)------------------------------------------------------------------------

1. If the answer for Q1is ICS or ICS+LABA, is there any circumstance in which you prescribe other drugs?

1-Yes

2-No

1. If the answer is ’yes’ for Q2, please specify the circumstances

---------------------------------------------------------------------------------------------------------------------

1. Do you recommend patients to take inhalation steroids continuously even if remain symptom free?

1-Yes

2-No

1. If ‘yes’ for Q 4 do you make any modification on the initial regimen?

1-Yes

2-No

1. If yes, what will you do?
2. Indicate regimen of your choice for the following category of persistence asthma? (with dose and frequency)

Mild persistent asthma--------------------------------------------------------------------------------------

Moderate persistent asthma-------------------------------------------------------------------------------

Severe persistent asthma----------------------------------------------------------------------------------

1. Do you think patient use prescribed ICS or ICS+LABA adequately?

1-Yes

2-No

1. If the answer for the Q8 is ‘No’, what do you think is the contributing factors?_________________________________________________________________________________________________________________________________________________________

**Thank You!!!**

50
